# Anti-*Toxoplasma gondii* activity of *Trametes versicolor* (Turkey tail) mushroom extract

**DOI:** 10.1038/s41598-023-35676-6

**Published:** 2023-05-29

**Authors:** Homa Nath Sharma, Jonathan Catrett, Ogechi Destiny Nwokeocha, Melissa Boersma, Michael E. Miller, Audrey Napier, Boakai K. Robertson, Daniel A. Abugri

**Affiliations:** 1 Department of Biological Sciences, College of Science, Technology, Engineering and Mathematics, Alabama State University, Montgomery, AL 36104 USA; 2 Microbiology Ph.D. Program, College of Science, Technology, Engineering and Mathematics, Alabama State University, Montgomery, AL 36104 USA; 3 Laboratory of Ethnomedicine, Parasitology and Drug Discovery, College of Science, Technology, Engineering and Mathematics, Alabama State University, Montgomery, AL 36104 USA; 4 Enterprise State Community College, Enterprise, AL USA; 5 Department of Chemistry, College of Arts and Sciences, Tuskegee University, Tuskegee, AL 36088 USA; 6 Department of Chemistry and Biochemistry, College of Science and Mathematics (COSAM), Auburn University, Auburn, AL 36849 USA; 7 Auburn University Research Instrumentation Facility, Harrison College of Pharmacy, Auburn University, Auburn, AL 36849 USA; 8 Present Address: The School of Dentistry (SOD) Doctorate of Dentistry Program, Meharry Medical College, Nashville, TN USA

**Keywords:** Antiparasitic agents, Drug screening

## Abstract

*Toxoplasma gondii* (*T. gondii*) infection continues to rise globally in humans and animals with high socioeconomic and public health challenges. Current medications used against *T. gondii* infection are limited in efficacy, safety, and affordability. This research was conducted to assess the higher fungi extract effect on *T. gondii* tachyzoites growth in vitro and possibly decipher its mechanism of action. Furthermore, we evaluated the extract's effect on human foreskin fibroblast viability. The methanol extracts of Turkey tail (TT) mushroom was tested against *T. gondii* tachyzoites growth using an RH-RFP type I strain that expresses red fluorescent protein throughout culture in a dose-dependent manner using a fluorescent plate reader. Similarly, we tested the effect of the extract on host cell viability. We observed that TT extract inhibited tachyzoites growth with a 50% minimum inhibitory concentration (IC_50s_), IC_50_ = 5.98 ± 1.22 µg/mL, and 50% cytotoxic concentration (CC_50s_), CC_50_ ≥ 100 µg/mL. It was discovered that TT extract induced strong mitochondria superoxide and  reactive oxygen species production and disrupted mitochondria membrane potential in *T. gondii* tachyzoites. Additionally, scanning electron microscopy depicted that TT extract and pyrimethamine (PY) caused a morphological deformation of tachyzoites in vitro*.* In conclusion, TT methanol extract made up of phytosterols, bioactive sphingolipids, peptides, phenolic acids, and lactones could be a promising source of new compounds for the future development of anti-*Toxoplasma gondii* drugs. Extracts were non-cytotoxic, even at higher concentrations.

## Introduction

Toxoplasmosis is an ocular, congenital, and neuroparasitic disease caused by a unicellular protozoan, *T. gondii. T. gondii* infection is globally prevalent in humans and animals^1–3^. *T. gondii* is considered as a neglected parasite, however, its infection in humans and animals contributes to public health, veterinary and socio-economic issues globally^1,4^. According to the CDC, the parasite can cause health-threatening complications in pregnant women, their fetuses, and people with compromised immunity^4^. In the US alone, it has been estimated that over 40 million people may have been infected with this successful zoonotic parasite^4^.

A recent study has shown that felids the cat family have a seroprevalence rate ranging from 35 to 75% globally^5^. The most worrisome issue associated with this parasite is its increasing presence in food-bound meat/ and products of animals and birds^1^. The most recommended drugs for the treatment of the fast-replicating form (tachyzoites) have been antifolates (i.e., pyrimethamine and sulfadiazine)^4,6,7^. However, this combination and others have been reported to have several toxicities issues, and treatment failure with the tachyzoites stage does not eliminate the slow-growing stage (bradyzoites)^6–8^ and are expensive for poor communities^9^. As a result of the increasing of these numerous challenges and the rising seroprevalence rates in both domestic and wild animals, birds, and the increasing emergence of diseases that could pose weak immunity in humans, finding new nutraceuticals or compounds for further development against parasite co-infection becomes ever so important and necessary.

Plants, algae, and fungi-based extracts and their metabolites have been reported to have promising sources of anti-parasitic compounds that could be developed further as effective and safe anti-parasitic agents^10–17^.

*Trametes versicolor* mushroom, which is classified as a polypore, has enormous medicinal uses globally^18^. Aside from its medicinal properties, it is rich in essential proteins, polysaccharides, sterols and their derivatives, bioactive sphingolipids, triterpenoids, and peptides, and has high antioxidants properties^15,16,19–23^. More specifically, *T. versicolor* has been reported to have antiparasitic properties (e.g. against *Leishmania* spp)^15,16^. Others have also reported its extensive antitumor properties^24,25^. However, no evidence existed about this mushroom effect on *T. gondii* tachyzoites growth in vitro*,* and its mechanism of action. The only study known in the field about fungus (mushrooms) anti-*T. gondii* activity is the study using the fungus *Trichoderma stromaticum*^26^. In this study, the researchers observed a decrease in *T. gondii* replication in vitro and an amelioration of experimental toxoplasmosis in vivo^26^. Based on our previous studies and others about this mushroom (TT) chemical properties, nutritional and health properties, and anti-*Leishmania* spp activity^11,15,23,27^, it attracted our interest to investigate its anti-*T. gondii* properties to decipher whether it could be developed into a safe and effective nutraceutical for zoonotic parasites managing in especially developing countries where these parasites' seroprevalences are very high, and the mushroom is abundantly found.

In this study, we investigated the effect of Turkey-tail mushroom extract inhibitory activity in *T. gondii* tachyzoites and assessed its cytotoxic effects. Also, we present a possible preliminary mechanism of action of the mushroom extract inhibitory activity against *T. gondii *in vitro*.*

## Materials and methods

### Mushrooms and extract preparation

The mushroom specimen [*Trametes versicolor* (Turkey-tail)] used in this study was collected in Tuskegee in 2018/2019 and was identified by Dr. Frederick Lafayette who is a mycologist affiliated with Tuskegee University, Tuskegee, AL, USA. The specimen of the mushroom is in Dr. Daniel Abugri’s laboratory at Alabama State University, Montgomery, Alabama, USA. The mushroom was extracted with methanol at 37 °C for 3 days and filtered using Whatman filter paper number 1. The residues were re-extracted three more times with 150 mL of methanol. All the filtrates were pulled together and dried using nitrogen evaporator/fume hood, yielding approximately 0.5 g crude extract. Crude extract was dissolved in DMSO at a rate of 2.54 mg/mL, and stored at − 20 °C until antiparasitic studies. The percent of DMSO in each dilution of extract for the biological assays was 0.1%.

### Parasites and cell cultures maintenance

Human Telomerase Reverse Transcriptase (hTERT) immortalized Human Foreskin Fibroblast (HFF) cells obtained from Dr. Silva NJ. Moreno (University of Georgia, Athens, GA) was maintained using Dulbecco’s Modified Eagle’s Medium (DMEM) without phenol red supplemented with 5% (v/v) fetal bovine serum (FBS), 200 nM L-glutamine and 1% (v/v) penicillin–streptomycin obtained from (Life Technologies, USA) and incubated at 37 °C with 5% CO2 and 95% atmospheric air. *T. gondii* (Type I strain, RH-RFP expressing red fluorescent protein in culture or RH-W, Wild type kindly provided by Dr. Silvia NJ Moreno, The University of Georgia, Athens, GA) was the strain type used in all the experiments. *T. gondii* tachyzoites were harvested by passing them through a 27-gauge needle followed by filtration with a 3 µm filter.

### Cell viability (cytotoxicity) assay

6 × 10^4^ hTERT cells/200 μL/well were seeded into the flat black bottom 96 well plates and incubated at 37 °C with 5% CO_2_ for 24 h^28^. Dead cells were removed by washing three times with 1X Phosphate Buffered Saline (1XPBS). The wells were refilled with 100 μL media and 100 μL of TT extract prepared in concentrations starting from 0, 0.78, 1.53, 3.06, 6.125, 12.5, 25, and 50 μg/mL. PY (Santa Cruz Biotechnology Inc., Dallas, Texas, USA) was used as the standard control with equivalent concentrations ranges tested in μg/mL. The plate was incubated at 37 °C with 5% CO_2_ for 72 h. Then, 10 μL of Alamar Blue (Abcam, Waltham, MA, USA) dye was added into wells and the plate was wrapped in aluminum foil and incubated at standard culture conditions for an hour. Next, plates were removed from the incubator and the fluorescence intensities of each well was measured at an excitation/emission wavelength of 485/535 nm, respectively. The percent host cell viability was calculated by comparing the fluorescence intensities of treated cells with the blank (medium with cells without drug) and the CC_50s_ values determined using graph Pad Prism software. Experiments were performed in quadruple (n = 4) with three independent wells each per experiment.

### *T. gondii* growth inhibition assay

To test the TT extract anti-parasitic properties, we seeded 6 × 10^4^ hTERT cells/200 μL/well as detailed in the cytotoxicity assay. After 24 h of adherence and confluence of cells, dead cells that were present were carefully removed by washing three times with 1X PBS. 6 × 10^4^ tachyzoites/100 μL of *T. gondii* RH-RFP Type I strain was added to each well and incubated at 37 °C with 5% CO_2_ for 3 h. Extracellular parasites were removed by washing with 1X PBS three times. Subsequent to washing, 100 μL of TT extract or PY was added in concentrations ranging from 0 to 50 μg/mL for TT extract, and 0–50 μM was used for PY as standard control. Media only was used as a negative control. Plates were incubated at 37 °C with 5% CO_2_ and read at 48 h using Tecan 200 F infinite microplate reader with excitation/ emission set at 560 nm/635 nm^28^, respectively. The percentage growth of the parasite was calculated with the formulae: (Average fluorescence of control parasitic growth-fluorescence of drug-treated parasitic growth/Average fluorescence of parasitic control growth) × 100. The percent inhibition was determined using the procedure reported by Huffman et al.^28^. Experiments were performed in quadruple (n = 4) with three independent wells each per experiment.

### Reactive oxygen species (ROS) production assay

1 × 10^5^ tachyzoites (100 μL) of *T. gondii* RH-Wild type I strain was added to each black flat bottom 96 well plates. 1.56 and 50 μg/mL of TT extract, 500 μM of hydrogen peroxide (H_2_O_2_) was used as a positive control and media only as a negative control were added into each well with the same volume of 100 μL media. The plate was incubated at 37 °C with 5% CO_2_ for 30 min. 10 μL of 5 μM of Cell ROX™ Purple reagent (Invitrogen Catalog # C10443) was added, the plate was wrapped with aluminum foil, and incubated at 37 °C for 30 min. Fluorescence intensities were measured using a Tecan 200 F infinite microplate reader with excitation set at 560 nm and emission at 635 nm. Experiments were performed in triplicates (n = 3).

### Mitochondrial superoxide production assay

1 × 10^5^ tachyzoites/100 μL of *T. gondii* RH-W (wild type) Type I strain was added to each well of the black flat bottom 96 well plates for assessing mitochondrial superoxide production by extracellular parasites. We then added 1.56 and 50 μg/mL of TT extract, 500 μM H_2_O_2_ was used as a positive control, and the media only as the negative control in the designated wells. Plates were incubated at 37 °C with 5% CO_2_ for 3 h. After incubation, 50 μL of 5 μM of MitoSOX™ reagent was added into each well, wrapped with aluminum foil, and incubated at 37 °C for 30 min according to the MitoSOX™ Red mitochondrial superoxide indicator (M36008) protocol provided by Invitrogen, USA. Fluorescence intensities for experimental wells were read using a Tecan 200 F infinite microplate reader with excitation set at 485 nm and emission at 535 nm. Experiments were performed in triplicates (n = 3).

### Mitochondrial membrane potential (MMP) assay

To tease out whether the high superoxide production exhibited by TT extract had any effect on *T. gondii* mitochondrial membrane potential, we used the cationic probe JC-1 (Thermo Fisher Scientific, Waltham, CA, USA) assay. Here, 1 × 10^5^ freshly purified tachyzoites from *T. gondii* RH wild-type I strain/50 μL media were seeded into a flat black bottom 96-well plate (Costar, Corning Inc., NY, USA). Then, 50 μL solution containing 1.56, and 50 μg/mL of TT as the experimental drug, 50 μM of Carbonyl cyanide m-chlorophenyl hydrazone (CCCP, from Alfa Aesar, Haverhill, MA, USA) as a positive control, and assay buffer (AB) as the negative control were added to the wells followed by incubation for 8 h at 37 °C with 5% CO_2_^28–31^. 10 μL of JC-1 was added to the wells, and the reaction plates were covered with aluminum foil and incubated for 45 min. Then, followed by centrifugation at 12 °C for 2000 rpm for 5 min, the supernatant was removed, and 100 μL assay buffer (Hanks Balanced Salt Solution (HBSS) without phenol red) was added to each well, followed by centrifugation at the same condition and removal of the supernatant. Next, 100 μL of assay buffer was added to suspend parasites in the solution. Parasites fluorescence was read at 560/635 nm for the JC-1 J-aggregates (590 nm) and at 485/535 nm for JC-1, J-monomers (529 nm) using a Tecan 200 F infinite microplate reader. The ratio of fluorescence values of J-aggregates to fluorescence values of monomers was calculated using the following formulae: Relative Fluorescence Units (RFUs) of J-aggregates (Red)/RFU of J-monomers (Green). Images were taken for each treatment group using an EVOS FL fluorescence microscope (Invitrogen Life Technologies, Carlsbad, CA, USA). Experiments were performed in triplicates (n = 3) and graphs were created.

### Sample preparation for scanning electron microscopy (SEM)

A confluent monolayer of Vero Cells (1 × 10^5^) per well of a 6-well plate (with coverslip on wells) was prepared. Then 1 × 10^5^ tachyzoites of *T. gondii* RH-W strains, along with 1.56 and 50 μg/mL of TT and 0.38 and 12.4 μg/mL of PY, were added into each well. The plate was incubated at 37 °C with 5% CO_2_ for 48 h. Cells were then washed with 1X PBS (3 times) and treated with 2.5% glutaraldehyde and kept at 4 °C overnight. Next, Vero cells containing tachyzoites were washed with 1X PBS (3 times), and 1% Osmium tetroxide was added and kept in the dark for 1.5 h. Cells were then washed with 1X PBS (3 times), dehydrated in a graded ethanol series (e.g. 50%, 70%, 90%, 100%), transferred to 100% acetone and then air-dried inside a fume hood^32^. Follow dehydration, the samples were sputter coated with gold and imaged using a Zeiss EVO 50 scanning electron microscope (Carl Zeiss Microscopy, Thornwood, NY USA.

### Phytochemical composition analysis using LC–MS/MS

Analysis was performed on a Vanquish UHPLC system (Thermo Fisher, USA) coupled with a quadrupole orbitrap mass spectrometer (Orbitrap Exploris 120, Thermo) with electrospray ionization (H-ESI) in positive and negative mode using Xcalibur software (V4.4.16.14). 10 μL of the TT extract (2.54 mg/mL) was injected onto a C18 column (ACQUITY UPLC® BEH C18, 1.7 µm, 2.1 × 50 mm, Waters) with a 200 μL/min flow rate of mobile phase of solution A (0.1% formic acid in 50% water 50% methanol) and solution B (50% acetonitrile and 50% isopropanol with 0.05% formic acid) beginning at 30%B to 50%B in 1 min followed by a linear gradient to 100%B in 13 min, held for 3 min, then returning to 30%B and 3 min of re-equilibration (total time of 21 min). The spray voltage was set at 3.5 kV in positive mode and 3.0 kV in negative mode. Methanolic TT extract were injected twice, one for each mode. The sheath gas was set at 30, aux gas at 20 and sweep gas 0 (all arbitrary units) with vaporizer temperature and ion transfer tube temperature 300 and 350 °C, respectively. The orbitrap resolution was set to 120,000 for MS and 15,000 for DDA MS/MS with 4 dependent scans with an intensity threshold of 20,000, auto dynamic exclusion, and a targeted exclusion mass list based on blank injections. EASY-IC was on for the MS scan with range 115–1000 Da. Collision energy was normalized and stepped at 10, 40, and 100 with the max injection time set to auto. Phytochemical analysis was performed at the Auburn University Research Mass Spectrometry Facility, Auburn, Alabama.

### Data processing

Samples were processed with Compound Discoverer 3.2 using the natural products and untargeted Metabolomics workflow with the Carotenoids Database, Human Metabolome Database, and LipidMAPS.

### Statistical analysis

The IC_50s_ and CC_50s_ were obtained using Graph Pad Prism software version 9.4.1. Comparison of means were carried out using Tukey’s multiple comparison test with the alpha value set to 0.05.

## Results

### In vitro anti-*T. gondii* activity and cytotoxicity

In this study, we report for the first time the anti-*T. gondii* inhibitory activity of Turkey-tail (*Trametes versicolor*) mushroom extract in vitro. The IC_50_ and CC_50_ of the TT extract were calculated to be IC_50_ = 5.98 ± 1.22 µg/mL, and CC_50_ ≥ 100 µg/mL respectively. To quality control our study, we used Pyrimethamine (PY) as a standard control which gave an IC_50_ = 4.98 ± 0.49 µM (1.22 ± 0.12 µg/mL), and CC_50_ ≥ 50 µM (12.44 µg/mL) (Table 1).

**Table 1:** 50% minimum inhibitory concentrations (IC_50s_) of TT (µg/mL) and PY (µM) against *T. gondii* and HFF 50% cytotoxic concentrations (CC_50s_) at 48 h’ interaction.

Compound (µg/mL)	IC_50s_	CC_50s_	SI
TT extract	5.98 ± 1.22 µg/mL	> 100 µg/mL	> 17
PY	4.98 ± 0.49 µM (1.22 ± 0.12 µg/mL)	> 50 µM (12.44 µg/mL)	> 10.0

Data are presented as means plus standard deviation from quadruple (n = 4) experiments performed in triplicate (n = 3) each. IC_50_-50% inhibitory concentration, CC_50_-50% cytotoxic concentration, and SI- selectivity index (CC_50_/IC_50_).

*T. gondii* inhibition curves for the 48 h have been presented in the supplementary Fig. 1.

To confirm that our effective parasites inhibition results obtained were due solely to the secondary metabolites found in the TT extract but not as a result of cytopathic effects on tachyzoites growth, we performed a host cell viability testing using the same concentrations used in the parasitic inhibition assay. The results are presented in Table 1. Interestingly, the TT extract and PY concentrations tested were not cytotoxic to host cells at the IC_50_ concentrations obtained for the *T. gondii* tachyzoites inhibition at 48 h (Table 1).

### Mitochondrial membrane potential (MMP) dysfunction

**Figure 1:**
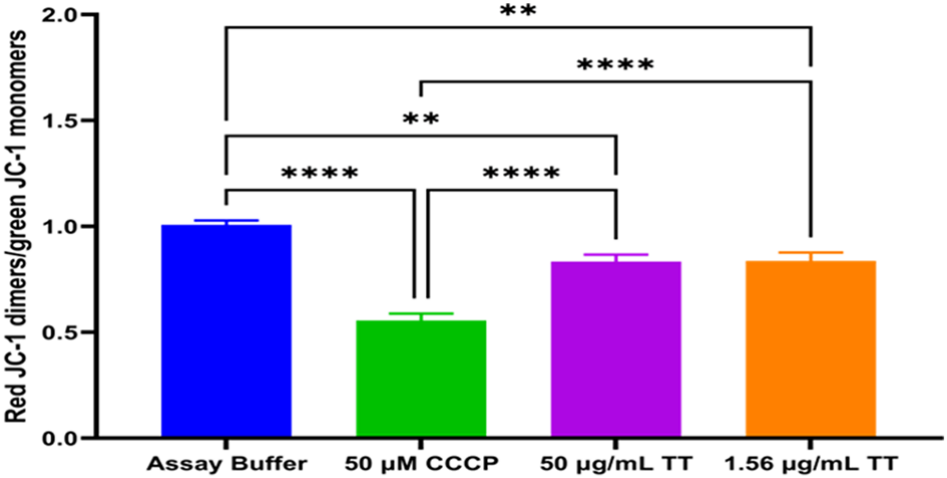
Ratio of JC-1-aggregate (560/635 nm) to JC-1 monomer (485/535 nm) fluorescence at of TT extract, assay buffer (negative control) and Carbonyl cyanide m-chlorophenyl hydrazone (CCCP) as positive control, **, **** indicates significant differences between the tested concentrations and the controls used with *p* < 0.01 and *p* < 0.0001, respectively.

Mitochondria are important organelles for *T. gondii* energy fulfillment, in this study, TT extract was found to induce a decrease in mitochondrial membrane potential (Fig. 1) implying that this might have affected the parasite survival and replication which are energy dependent. There were statistical differences between the negative control (assay buffer only) versus 1.56 µg/mL TT extract (*p* < 0.0001), assay buffer only versus 50 µg/mL TT extract (*p* = 0.0038), and assay buffer only versus positive control (CCCP) (*p* < 0.0001). Furthermore, there was a statistical difference between the mitochondrial membrane potential disruption using the CCCP (positive control) and the selected concentrations of the TT tested with *p* < 0.0001 (Fig. 1).

### Mitochondrial superoxide (MitoSOX) production

**Figure 2:**
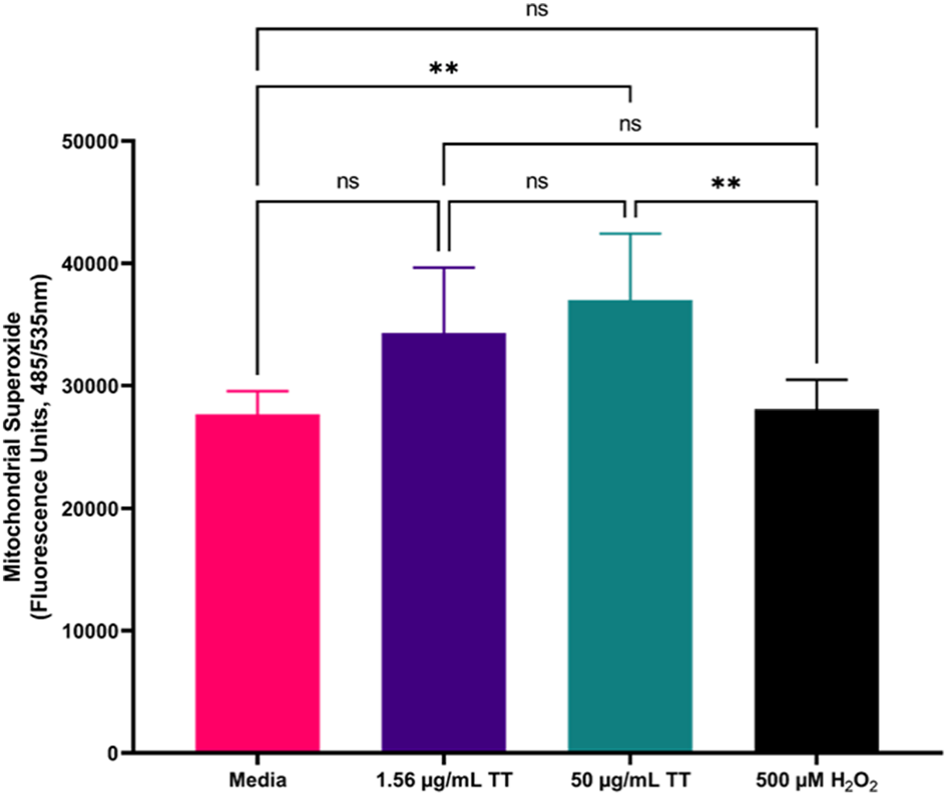
Effect of Turkey-tail mushroom extract on *T. gondii* tachyzoites mitochondrial superoxide production. ** indicate significance with *p* < 0.01. The plate reader used the excitation/emission spectrum of 485/535 nm. *ns* no significance difference.

TT extract was found to cause mitochondrial superoxide production in *T. gondii *in vitro (Fig. 2).

Significantly, the mushroom extract caused tachyzoites mitochondria superoxide production with a strong statistical difference between the negative control (media only) *p* < 0.01, and the positive control (H_2_O_2_) *p* < 0.0001.

### Reactive oxygen species (ROS) production

**Figure 3:**
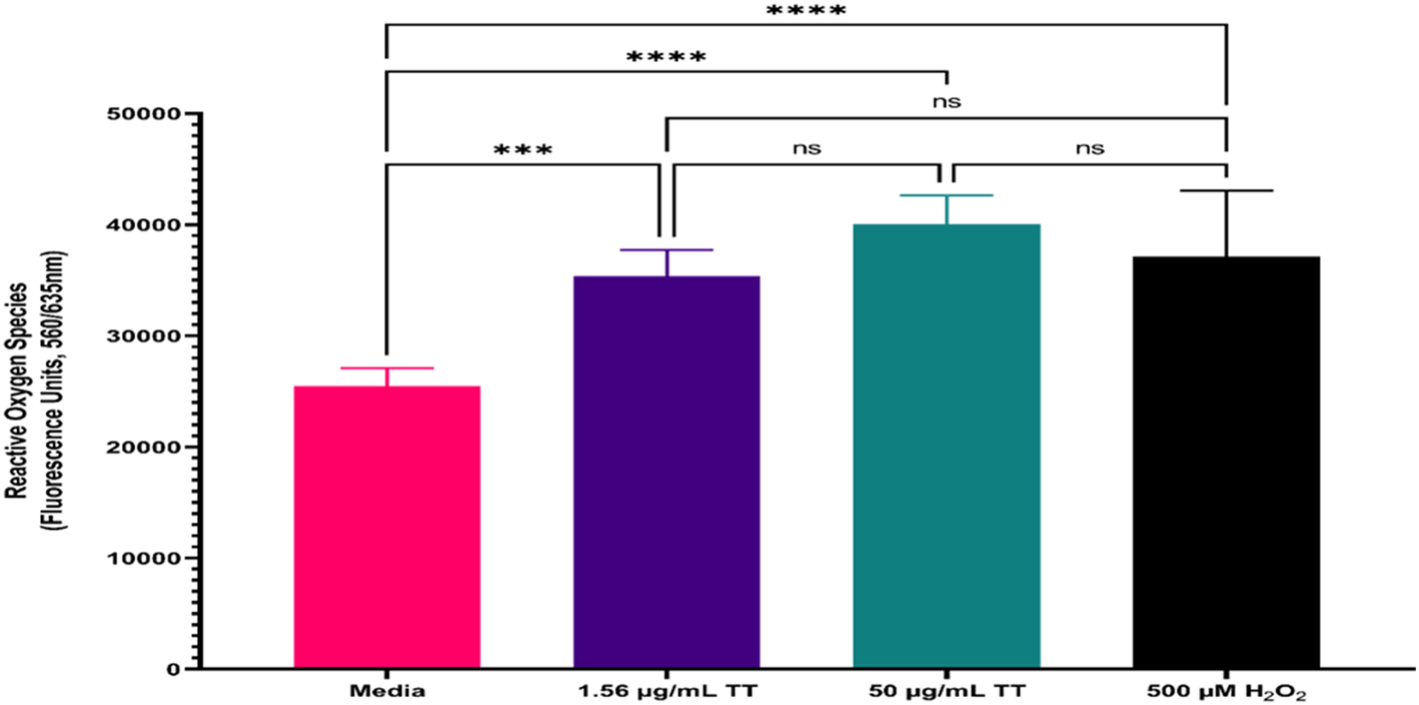
Effect of Turkey-tail mushroom extract on *T. gondii* tachyzoites Reactive Oxygen Species (oxidative stress) production. *** and **** indicate significance with *p* < 0.001 and *p* < 0.0001, respectively. *ns* no significance difference.

The 50 μg/mL methanolic extract of TT was found to cause higher production of reactive oxygen species in tachyzoites as compared to media (negative control) in vitro (Fig. 3) with *p* < 0.0001. Similarly, the positive control (500 μM of H_2_O_2_) ROS production was statistically different compared to the media (negative control) treated tachyzoites ROS generation (*p* < 0.0001). There was a significant difference between the 1.56 μg/mL and the media (negative control) with *p* < 0.001. Contrarily, the ROS produced by both the lower and higher concentrations of the TT extract was not concentration dependent.

### Intracellular *T. gondii* tachyzoites morphological alteration

**Figure 4:**
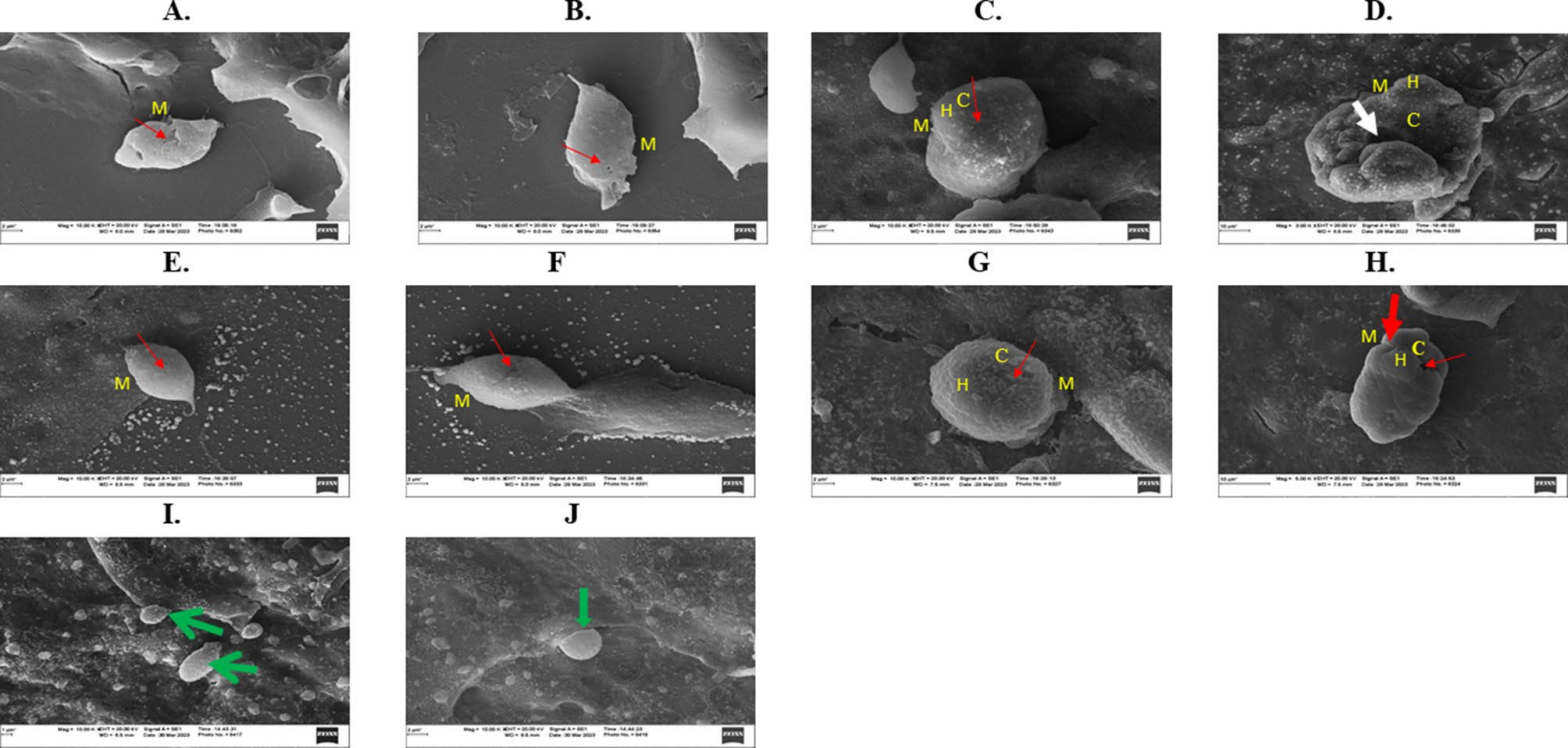
Scanning Electron Microscopy (SEM) photomicrograph of *T. gondii* tachyzoites treated with 1.56 μm (0.38 μg/mL) of PY (**A**, **B**), 50 μm (12.4 μg/mL) of PY (**C**, **D**), 1.56 μg/Ml of TT (**E**, **F**), 50 μg/Ml TT (**G**, **H**) and media as negative control (**I**, **J**). *H* hook, *C* cellular disintegration/collapse, *M* membrane disruption/perforations; and long red arrows indicate holes, short white arrow indicates wrinkles, and short red arrow indicates dimples. Green arrows indicate the normal crest-like smooth, and regular size of a typical intracellular tachyzoites.

Scanning electron microscopy (SEM) was used to assess the effect of TT and PY against the outer morphology of the *T. gondii* tachyzoites inside the Vero cells. Treated tachyzoites were relatively swollen with hooks (extensions). The micropore (primary portal for normal endocytosis), along with its crescent shape, has been transformed with an unusually collapsed, perforated, and disintegrated shape characterized by more holes, deep pimples, and wrinkles (Fig. 4A–H). These effects were more pronounced at higher concentrations of drugs (50 μg/mL of TT and 12.44 μg/mL of PY) used compared to the lower concentrations of the drugs (1.56 μg/mL of TT and 0.38 μg/mL of PY) (Fig. 4A–H). The control (untreated) tachyzoites were found to have a typical crescent shape characterized by smooth, regular, and complete outer surfaces (Fig. 4I–J). The pattern of the result was consistent with the previous result regarding the effect of the compound found to inhibit *T. gondii*^32^.

### Phytochemical compositions of methanolic TT extract

Mass spectrometry analysis revealed that the TT extract contained phytosterols, ergostanes, lanostane, peptides, fatty acids, triterpenoids, phenolic acids, lipids (phospholipids and sphingolipids), and vitamins (sup Table 1).

## Discussion

Traditionally, toxoplasmosis is treated using antifolate inhibitors (pyrimethamine and sulfadiazine)^4,6,7^. However, these drugs and the second-line drugs used for treatment have serious clinical side effects on patients and require repeated doses for parasite clearance^6–8^. Additionally, these drugs do not inhibit the tissue cyst (bradyzoite) form of *T. gondii*^6–8^. These current medication drawbacks necessitated the discovery of novel compounds that could treat parasitic infections in humans and animals.

The current study showed that TT extract effectively inhibited *T. gondii* growth intracellularly with minimal cytotoxic effects (Table 1). Our IC_50s_ value (5.98 µg/mL) was consistent with some of the previous works reported in *T. gondii* (RH-YFP) type I strain using natural extracts and compounds derived from *Sorghum bicolor* with IC_50s_ ranging from 0.36 to 20.38 µg/mL^21,22^. However, our results differed from the in vitro experiments reported in *Leishmania* spp^15,16^*.* The possible reasons for the disagreement could be partly due to the solvent of extraction, the composition of TT extract, and the type of protozoan tested.

The typical IC_50_ value for PY against *T. gondii* tachyzoites growth is between 0.0025 to 1.05 µg/mL^28,33,34^. However, our value for PY was high. The high IC_50_ value reported for the pyrimethamine could have been attributed to our observation of the drug precipitation in the culture medium and might have caused less absorption by tachyzoites cells and thus impeded its usual potency against the parasite's growth. Noteworthy in this study was the selectivity index for the TT extract which was calculated to be > 17 and that of PY to be > 10. This implies that TT extract will have a broad spectrum in inhibiting *T. gondii* tachyzoites in vitro than causing a cytotoxic effect as seen in the primary drug (PY) reported in the literature^12^.

Interestingly, more compounds were found in this current work compared to previous studies using *n-*Hexane Leliebre-Lara et al.^15^, Leliebre-Lara et al.^16^. The different compositions of chemicals observed could be attributed to seasonal and geographical parameters^23^. Also, in previous studies by Leliebre-Lara et al.^15,16^, the n-hexane and ethanol extracts were fractionated, and this might have removed some of these components found in our mass spectrometry data using the methanolic extract. Additionally, in our previous work, we discovered that methanol was the best solvent for extracting large classes of secondary metabolites in mushrooms^27^.

Our phytochemical data confirmed some of the previous studies of *Trametes versicolor* chemical composition to include ergosterol, 5α, 8α-epidioxy-22E-ergosta-6, 22-dien-3β-ol and trametenolic acid B in TT extracts obtained using *n*-Hexane^15,16^.

Furthermore, these compounds have been reported to have anti-*Leishmania* activities Leliebre-Lara et al.^15^, Leliebre-Lara et al.^16^. Therefore, the anti-*T. gondii* properties observed in TT extract could be attributed to the presence of bioactive lipids, fatty acids, peptides, organic acids, lactones, ergosterol, 5α, 8α-epidioxy-22E-ergosta-6, 22-dien-3β-ol, trametenolic acid B and phenolic compounds^11,14–16^. The compounds reported here have been found to perturb cellular membranes, cause growth arrest in cancer cells, cause mitochondrial membrane potential disruption, and radical productions^35,36^.

Our data are supported by previous studies using synthetic sterols analogs (22, 26-azo sterol and 24, 25-(R, S) epiminolanosterol) that showed an effective inhibition of *T. gondii* tachyzoites growth in vitro^37,38^. These sterols are known to inhibit sterol biosynthesis enzyme 24(25)-sterol methyltransferase (SMT) and have been documented to have strong inhibitory activity against several protozoan parasites, including *T. gondii*, *Trypanosoma cruzi, Leishmania* spp^37–41^ and *Giardia lamblia*^42^. Although *T. gondii* does not contain the sterol biosynthetic pathway^43^ that these sterols target in other protozoa, it has been discovered to cause mitochondria distortion, plasma membrane disruption, and eventually parasites death^38^. The mitochondrial membrane disruption observed by JC-1 assay in this current work supported the previous report by the following researchers^37,38^, about sterols' ability to disrupt parasites’ mitochondria leading to ineffective oxidative phosphorylation and eventually parasites death. Significantly, the SEM data showed that TT and PY treatments had morphological defects on intracellular tachyzoites. Specifically, tachyzoites were observed to be swollen in size with hooks (extensions). Also, the micropore (primary portal for normal endocytosis), along with its normal crescent shape, was observed to portray an unusually collapsed, perforated, and disintegrated shape with more holes, deep pimples, and wrinkles (Fig. 4A–H). These alterations were more pronounced at higher concentrations of drugs (50 μg/mL of TT and 12.44 μg/mL of PY) treatment compared to the lower concentrations of the drugs (1.56 μg/mL of TT and 0.38 μg/mL of PY) used (Fig. 4A–H). This observation was consistent with previous findings regarding the effect of certain type of compounds found to inhibit *T. gondii* growth^32,37,38^. This observation confirmed that the TT extract induction of high ROS, and MitoSOX productions in tachyzoites led to a remarkable disruption of the mitochondrial membrane potential (Figs. 1, 2, 3). Additionally, these may have caused the mitochondrial deformities and structural alteration found in tachyzoites, which need to be confirmed with further experiments.

Other studies have also shown that ergosterol peroxide (EP) which was identified in our current study affects *Entamoeba histolytica* growth^44^; anticancer activity^24^, antitrypanosomal^17^, anti-leishmania spp^15,16^, and antiviral properties^45,46^. EP antiviral (SARS-COVID) mechanism of action was reported to be associated with cell growth arrest, causing oxidative stress, attachment, entry, and blocking of early, and middle stages post-entry stage^45,46^. Also, compounds such as 3-beta-Hydroxylanosta-8, 24-dien-21-oic acid [trametenolic acid (TAB)] have been reported to inhibit cancer cell proliferation through the inhibition of H^+^/K^+^-ATPase activity^47^. This implies that TAB might have also affected *T. gondii* H^+^/K^+^-ATPase activity and possibly Ca^2+^ signaling which play a crucial role in *T. gondii* lytic cycle operation intracellularly. This conjecture requires further studies. Furthermore, in the literature ursolic acid has been reported to inhibit MCF-7 cells, causing apoptosis of cells leading to G1 arrest in cancer cells death^48^. This compound presence in the TT extract might have also contributed to parasite proliferation arrest, ROS and the MitoSOX induction resulting in tachyzoites death.

Another compound that was discovered in our TT extract fingerprinting analysis was Enniatin B, which has been documented to have antibacterial, antihelmintic, antifungal, herbicide, and insecticidal activity^49^. This compound mechanism of action is through the induction of high ROS signaling, apoptosis, DNA fragmentation, and K^+^/Ca^2+^ channel modulation^49^.

Taken together, all the unique chemical profiles of TT extract and the known biological activities of some of these compounds discovered, it is believed that TT has potent compounds that could be explored further to validate their individual and combination activity, especially in those compounds known to exert similar biological mechanisms of action.

## Conclusion

In summary, our results about the inhibitory activity of the TT extract, its less cytotoxic effects and the chemical profile showed that there are polar compounds that could be isolated for further screening and chemical modification for in vivo studies. Also, the TT extract possible mechanism(s) of action against *T. gondii* death was through high ROS and MitoSOX production resulting in high parasite stress, mitochondrial membrane depolarization, morphological deformities, and eventually mitochondria collapse.

## Supplementary Information

Supplementary Information 1.

Supplementary Information 2.

## Data Availability

The raw data used for the graphs are available upon request from the corresponding author. The chemical fingerprinting of the TT extract has been provided in the supplementary Table 1.
