# Supplementary figures and images for: Anti-Toxoplasma gondii activity of Trametes versicolor (Turkey tail) mushroom extract
Source: Sci Rep. 2023 May 29;13:8667. doi: 10.1038/s41598-023-35676-6 (PMC10225767; doi:10.1038/s41598-023-35676-6)

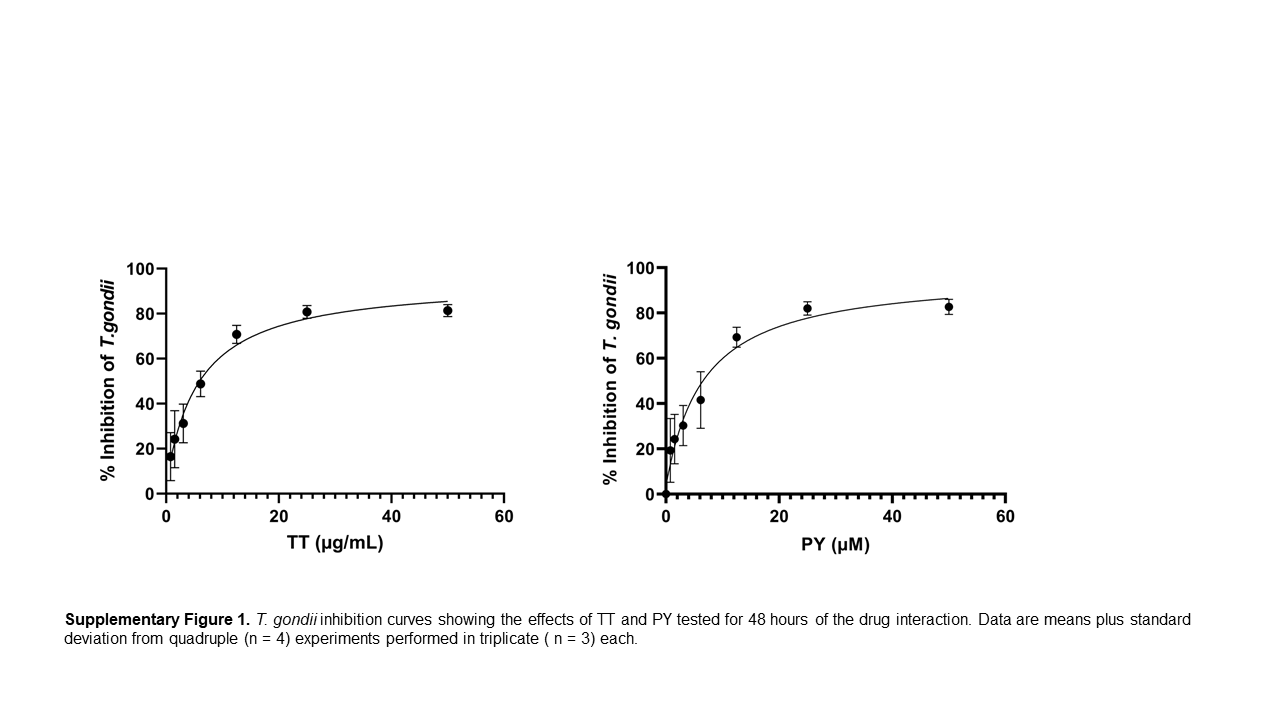

Supplement: Supplementary file 1 — Supplementary Information 1. [file 41598_2023_35676_MOESM1_ESM.tif]
